# How well do UK assistantships equip medical students for graduate practice? Think EPAs

**DOI:** 10.1007/s10459-023-10249-4

**Published:** 2023-06-22

**Authors:** Ruth Kinston, Simon Gay, R. K. McKinley, Sreya Sam, Sarah Yardley, Janet Lefroy

**Affiliations:** 1 School of Medicine, Keele University, Clinical Education Centre, University Hospital of North Midlands, Newcastle Road, Staffordshire, ST4 6QG UK; 2 University of Leicester School of Medicine, Leicester, UK; 3 Keele University School of Medicine, Keele, UK; 4 Marie Curie Palliative Care Research Department, University College London, London, UK; 5 Central & North West London NHS Foundation Trust, London, UK; 6 School of Medicine and Faculty Lead for the Health Professionals Education Research Theme, Keele University, Keele, UK

**Keywords:** Education, medical, Students, medical, Entrusted professional activities, Realist approach, United Kingdom

## Abstract

The goal of better medical student preparation for clinical practice drives curricular initiatives worldwide. Learning theory underpins Entrustable Professional Activities (EPAs) as a means of safe transition to independent practice. Regulators mandate senior assistantships to improve practice readiness. It is important to know whether meaningful EPAs occur in assistantships, and with what impact. Final year students at one UK medical school kept learning logs and audio-diaries for six one-week periods during a year-long assistantship. Further data were also obtained through interviewing participants when students and after three months as junior doctors. This was combined with data from new doctors from 17 other UK schools. Realist methods explored what worked for whom and why. 32 medical students and 70 junior doctors participated. All assistantship students reported engaging with EPAs but gaps in the types of EPAs undertaken exist, with level of entrustment and frequency of access depending on the context. Engagement is enhanced by integration into the team and shared understanding of what constitutes legitimate activities. Improving the shared understanding between student and supervisor of what constitutes important assistantship activity may result in an increase in the amount and/or quality of EPAs achieved.

## Introduction

The period of transition from learner to practitioner is challenging for learners, educators, employers and regulators. Learners have long felt unprepared, educators are unsure how to prepare their learners, employers are unsure how to support their new employees, and regulators have been challenged as to how to manage the process (Monrouxe et al., 2017).

Research on the transition from learner to (semi) autonomous practitioner has focused the preparation of the learner for practice by, for example, wholesale curricular reform (Cave et al., 2007) or more focused innovations such as simulation of practice or reducing the ‘step’ between learner and practitioner through ‘graduated exposure’ to practice with sub-internship or assistantship experiences as learners (Braniff et al., 2016; Crossley & Vivekananda-Schmidt, 2015; Hawkins et al., 2015; Reddy et al., 2014). While such innovations have been beneficial and widely adopted through both passive diffusion and the force of regulation (GMC, 2022), learners remain variably prepared for practice both within and between institutions (Goldacre et al., 2010; Monrouxe et al., 2017).

In common with our previous work (Lefroy et al., 2017; Yardley et al., 2020), this study utilises data from a multiphase ethnographic approach based upon a 3-stage conceptual framework, developed from socio-cultural learning theory. We mapped our investigations, and collected data from multiple sources, for each of the three stages of transition: anticipation (the undergraduate experience of preparation for practice), the lived experience (newly qualified doctor undergoing transition) and reflection on transition (looking back on experiences of early clinical practice). Our first study shed some light on why preparation for practice varies. By realist evaluation of the whole dataset we produced a midrange theory proposing that new doctors are able to face and learn from ‘firsts’ in practice when their internal sense of ability and responsibility and their colleagues’ external expectations of them cohere. Therefore, in ideal circumstances, giving and acceptance of increasing responsibility should be a gradual process, according to the level of support needed with appropriate challenges which enable new doctors to confirm their abilities (Lefroy et al., 2017).

Our second study used a storytelling analytic tool (Labov, 1997) with focus on meaning constructed by participants, to provide a new lens through which to view all data with a responsibility code, sub-theme or theme cross-matched with all data coded to an identity code, subtheme or theme. The recurring narrative structures identified the centrality of participation with responsibility to the process of identity formation and acceptance of the responsibilities of being a doctor (Yardley et al., 2020).

We suggested that successful transition requires a supported period of identity reconciliation during which responsibility may feel burdensome. There is a fine line between too much and too little responsibility and this line varies between and within learners.

Delegation of authority to transitioning learners whilst maintaining true oversight therefore needs to be encouraged. Learners need support to integrate these experiences and to facilitate their transition from student to qualified doctor. One possible way of enriching assistantships to better promote these goals would be to deploy Entrustable Professional Activities (EPAs).

First described in the mid-2000s, EPAs are designed as a bridge between competency frameworks and clinical practice (Ten Cate et al., 2017). They are defined as a…

*“…unit of professional practice that can be entrusted to a sufficiently competent learner or professional,… [which] requires proficiency in multiple competencies simultaneously.”*

(Ten Cate et al., 2015).

They closely align with clinicians’ daily work and together constitute the mass of critical elements that operationally define professional practice and expectations of performance including at entry level to the profession (Bramley & McKenna, 2021; Meyer et al., 2019). EPAs are underpinned by educational theory as a mechanism to support individuals becoming more independent practitioners (Chen et al., 2015; Dornan et al., 2007; Lave and Wenger 1991). Educational effects are linked to receiving immediate feedback after activity and judging a trainee’s capability to perform clinical duties over time, and from multiple sources (Meyer et al., 2019). They may also encourage learners to strive for excellence and personal progression, goals which may have been devalued by competency-based learning (Brooks, 2009; Frank et al., 2010; Lobst et al., 2010; Ten Cate & Scheele, 2007). They therefore have attractions as a framework within which to encourage medical students to both participate in clinical work and to accept responsibility for their work, both of which are integral to accepting the identity of a doctor in waiting (Cruess et al., 2015).

The literature describing the use of EPAs in undergraduate medical education has bloomed. However, only the minority present empirical research about their application in practice (Bramley & McKenna, 2021; Meyer et al., 2019; Pinilla et al., 2021). Several studies report using EPAs to define educational outcomes or expectations of performance at entry level to the profession (Bramley & McKenna, 2021). Flexibility in attaining competencies and achieving entrustment when ‘trustworthy’ is integral the adoption of EPA frameworks (Gruppen et al., 2018) though there remains a paucity of evidence comparing entrustment outcomes to other assessment methods (Meyer et al., 2019; Pinilla et al., 2021). There are concerns that increased entrustment on the EPA scale may be influenced by other factors, rather than increased ability (Bramley & McKenna, 2021; Pinilla et al., 2021; Shorey et al., 2019).

Effective EPA assessment relies on gathering multiple independent professional observations under varying conditions (Linsenmeyer et al., 2018). Graduates who felt prepared to perform many core EPAs under indirect supervision also report an easier transition to postgraduate year-1 practice (Obeso et al., 2021). However, currently multiple tensions exist in the implementation of EPAs in UME and at present they are not recommended for use in high stakes graduation decisions (Geraghty et al., 2021). Despite these issues we believe that EPAs may still be effective in UME since they create a framework for the graduated giving/receiving responsibility. They also improve the content, reliability, and quantity of feedback (Kuehl & Spicer, 2022). However, engagement with activities depends on many factors including the supervisor’s acquaintance with the leaner and their year of study (Sterkenburg et al., 2010). It may be affected by the mismatch between self-appraisal of entrustability with that of their supervisor(Soukoulis & Gusic, 2018). It has also been suggested that students need smaller more targeted (nested) EPAs, which contribute towards the achievement of a more generic EPA, especially during shorter, specialty specific placements (Pinilla et al., 2021). Students may also perceive entrustment less relevant in some settings since activities were conducted by residents (Pinilla et al., 2021).

It is therefore important to know whether and how students engage with recognisable EPAs during their assistantships (sub-internship): if they don’t it is unlikely that EPAs will form a useful framework to support transition to, and preparedness for, practice.

In the third paper in the series, we therefore focus our analysis of our large data set of previously unused log sheets and audio diaries, interviews, and focus groups on the natural occurrence of EPAs and the opportunities for entrustment decisions (Sterkenburg et al., 2010; Ten Cate, 2013), ad hoc or otherwise, that arose while our participants were final year medical students in assistantships at a number of UK medical schools.

### Research questions

- What EPA opportunities do assistantship placements present to medical students?
- What are the mechanisms by which students are entrusted to engage with professional activities in order to become more equipped for practice?

### Context

The assistantship at Keele is undertaken during the final year of the 5-year medical degree after completion of the final summative knowledge assessment. The assistantship period is 30-weeks duration and comprised of two, 15-week blocks, one in primary care and one in secondary care (5-weeks of medicine, surgery and acute care).

During assistantship placements students should be fully integrated within a clinical team and should be responsible for carrying out specified duties under appropriate supervision (GMC, 2022). Assistantships aim to prepare students to commence foundation practice (a compulsory 2-year internship), ahead of commencing core specialty training (residency). Whilst no EPA framework is explicitly utilised, during each block the assistantship student is encouraged to assist in the supervised delivery of care to patients. Students are also required to complete a procedural skills logbook consisting of regionally agreed practical procedural, life support and prescribing skill competencies that an undergraduate medical student should achieve prior to graduation (General Medical Council, 2019). The student is required to collect evidence (supervisor’s sign-off) of any skills they complete in clinical practice. Doctors in training locally are drawn from various UK medical schools.

## Methods

### Methodology

To answer the research questions addressed in this paper we conducted a new realist orientated analysis of two datasets; one being the autoethnographic log sheet of students learning activities and the other being talk in interviews about medical school learning and what made it work well or not. Realist evaluation asks the question “What works for whom under what circumstances and how or why?” (Wong et al., 2012). It is an ideal research method for studying complex social programmes which have a theoretical mode of working (such as assistantships in undergraduate medical education) but a variety of outcomes in practice for different individuals and in different settings. Realist methods aims to take the initial programme theories about how the programme should work and test them in the experience of participants who explain what has actually worked for them and why. By identifying recurring patterns of association between contextual factors and outcomes and the mechanisms by which these come about as explained by participants, the initial programme theories are developed in a useful way by the addition of context. Those wishing to run the same programme (medical student assistantships, for example) in another setting can use the detailed theories about why it should work (or not) for whom and in what contexts to adjust their own programme to be more likely to work (while remaining alert to new refinements of the theories based on further experience). Our initial programme theory postulated that entrustment and learning the jobs of the junior doctor were important elements of assistantship. In this study our investigation examined what was really happening in students’ autoethnographic data against the EPA framework.

*Ethical approval was granted by Keele University School of Medicine ethics committee on 4.9.13.*

### Recruitment and participation

We used data from two groups of participants:

1. In 2013–2014, all Keele final year students (n = 124) were invited to participate in a study of how their senior assistantship prepared them for practice. Participants were also asked to consent for follow-up into their first year in qualified clinical practice, Foundation Year 1, (FY1) in August 2014.

2. We recruited participants by email invitation to all doctors working in foundation (postgraduate years 1 and 2) or specialty training (postgraduate year 3 or later) posts in our local teaching hospitals. Participants were mostly from other UK medical schools but included some Keele graduates.

### Data generation

*Data from participants recruited while Keele students*: ‘Snapshots’ of students’ experiences of anticipation of and transition to practice with their immediate reflections were captured using learning logs and audio diaries. Participating students completed these each working day during specific weeks of their assistantship (fourth week in each of three 5-week hospital rotations, or week 4, 9, or 14 of the 15-week GP assistantship). The logs listed all their activities each day that week and categorized any learning which occurred (i.e. whether they observed another professional/ practised themselves/ listened to a talk about or read about/discussed). Any activity could be in one or all of these categories. Their audio diaries captured their immediate reflections on new, interesting and significant learning experiences. We also sought to involve these participants in analysis of their anticipation and transition using interviews in which their own-recorded data was discussed. Researchers who were medical school staff but not directly involved in the students’ education, conducted the face-to-face interviews. Interviews took an approach consistent with realist interviewing, asking students to explain why their learning was valuable or lacking. For example, students were asked “How is your medical school experience so far preparing you for these transitions? What transitional doctor roles are you being given, what activities are helpful? (Appendix X–on-line). Those who consented to further follow up were asked to make voicemail audio diaries during their first weeks of their first post after qualifying as a doctor, with a second interview conducted 3 months later when they had moved into their next post.

**Table 1:** Timeline of participant activities and data collection

Timeline	2013—2014				2015	
	Sept to May				Aug to Dec	Jan
Final year medical student participants n = 32	Learning logs (n = 32) Audio diaries (n = 27) Written diary (n = 1) Interviews (n = 24)			Consented to be followed through the transition n = 14	FY1 Voice-mail diaries n = 11	FY1 interviews n = 6
Postgraduate doctor participants n = 70 (57 FY1, 13 CT2)	FY1 individual interviews n = 7	Four FY1 focus groups n = 29	Two CT2 focus groups n = 13			Four FY1 focus groups n = 28
KEY to quotes						
Respondent	Type of data and individual identifier					
Student (in the UK pre-qualification these are undergraduate medical students)	Individual identification numbers are given as student IDxx e.g. student ID29					
	Data source is bracketed e.g. (interview)					
	Interview Individual identification numbers are given as FYIDxx					
Foundation year (FY) (in the UK, these are newly qualified doctors, FY1—first year post qualification, FY2—second year post qualification. Intern would be the international equivalent)	Focus groups Identified by Site (1–3) and Group e.g. 2 Individual identification numbers are given as FGIDxx					
CT2: General internal medicine core trainee year 2 doctors, 4th postgraduate year (Residents)	Focus groups Identified by site (4) and group Individual identification numbers are given as FGCTxx					

### Analysis of student activity logs

Student activities were coded by two researchers (SS and JL) to EPA type (Netherlands 2015–2017 Entrustable professional activities for curriculum Utrecht Plus (CRU +) (Ten Cate et al., 2018) or other (non-EPA) activity, together with level of supervision which was coded using the Utrecht supervision scale for medical schools (Chen et al., 2015) (Table 2).

Student log sheets distinguished between learning by observing (coded as level 1), by doing (coded as level 2 to 3), or by teaching (level 5). Students also logged learning by discussion, by reading or listening to a talk. These did not map to the supervision scale but were coded as two additional types of learning (‘Discussion’ or ‘Reading or listening’).

**Table 2:**
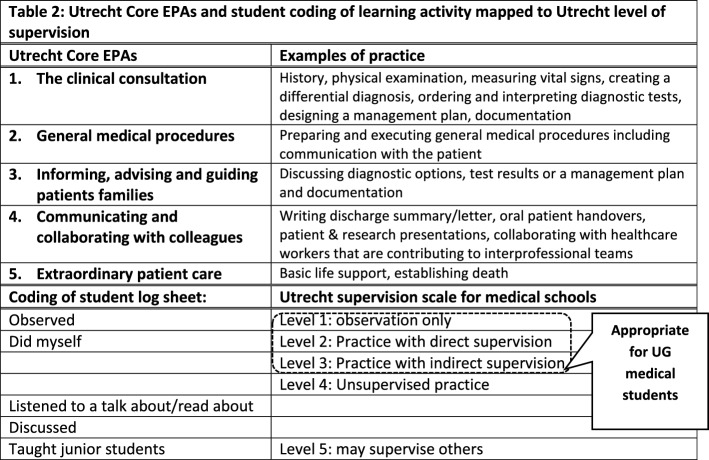
Utrecht Core EPAs and student coding of learning activity mapped to Utrecht level of supervision

*Data from medically qualified participants recruited post-graduation*: We used interviews and focus groups to generate data. Focus groups were arranged as an optional add-on to training days. Audio recordings were transcribed and anonymised. The transcripts were uploaded into QSR NVivo version 9 for analysis. Transcripts were initially coded thematically using a framework the researchers developed by constant comparison while data was being collected (Lefroy et al., 2017).

**Fig. 1:**
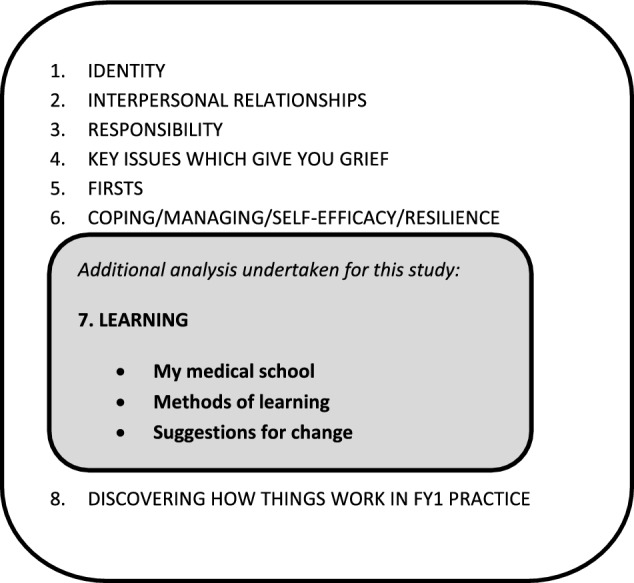
Coding Framework Used Initial Analysis of The Transitions Study

This coding framework is outlined in Fig. 1. The coding basket for ‘learning’ with sub-codes – ‘learning—my medical school’ ‘methods of learning’ and ‘learning—suggestions for change’ were analysed as the data for this study.

### Analysis of student and doctor talk about medical school preparation

Our realist analysis started with a programme theory which we wanted to develop in more detail in this study. We specifically wished to explain how the assistantship might affect participation in professional activities and in preparing participants for practice. The initial programme theory for this whole research project looking at the lived experiences of transition has already been developed by our study of junior doctor ‘firsts’ (Lefroy et al., 2017). Our final programme theory from that study, in summary, was “Helpful interventions in preparation (context) were opportunities for rehearsal and being given responsibility as a student in the clinical team. Building self-efficacy for tasks was an important mechanism. During transition the key contextual factor was the right sort of support from colleagues” (Lefroy et al., 2017). In this third study we focus our realist analysis on these opportunities for rehearsal and responsibility in medical school. Using the terminology of EPAs, the initial programme theory for this analysis of our data about medical school preparation was that medical student assistantships work by giving students the opportunity (a Context – C1) to experience the tasks of the qualified doctor under supervision (EPAs) (a Mechanism – M1) so that they can cope with the tasks with a reduced level of supervision when they meet them as junior doctors (an Outcome – O1). The more opportunities students get (C + M), the more prepared they feel (O), even if this rehearsal is sometimes in simulation or mental preparation (C).

**Fig. 2:**
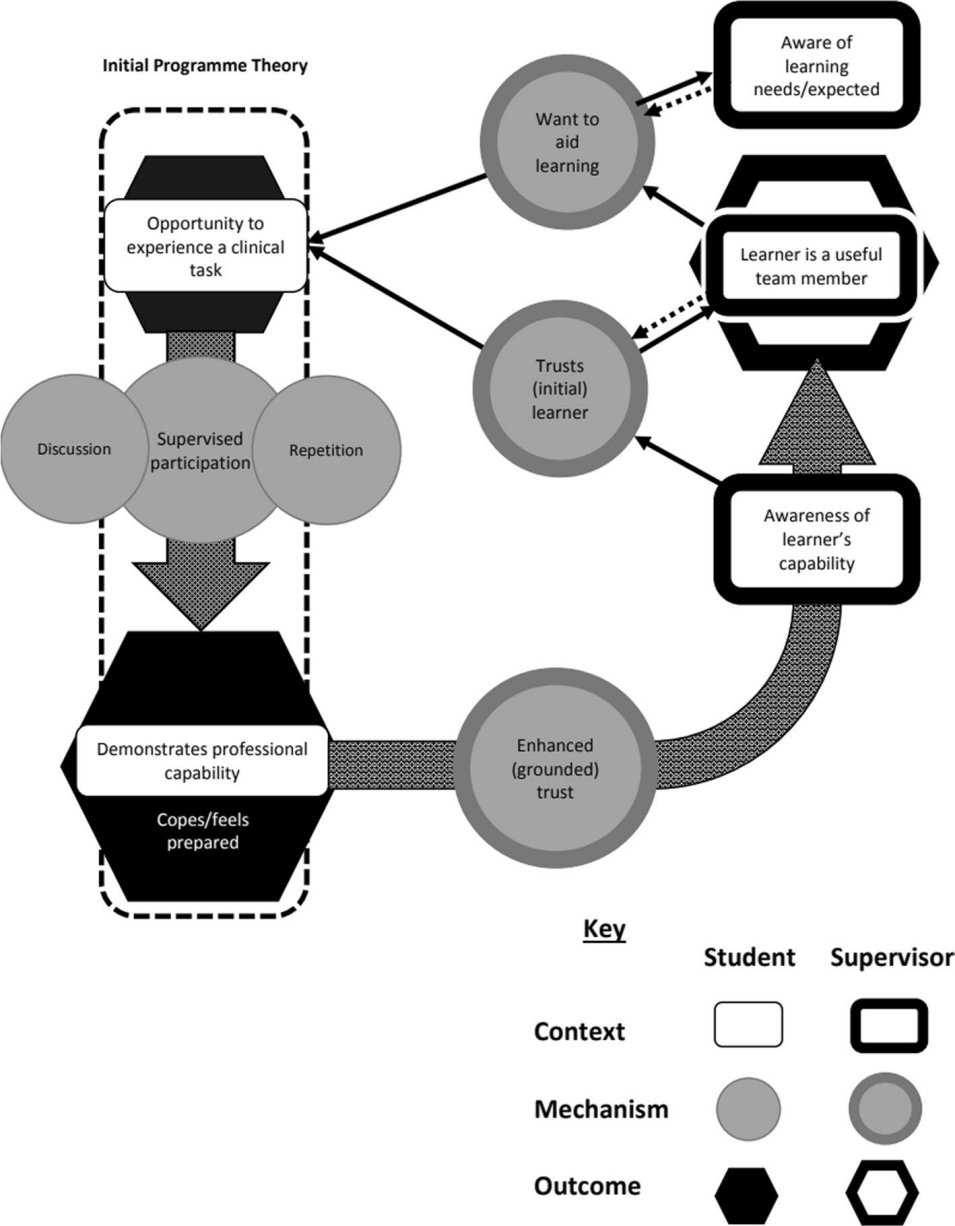
How EPAs work in medical student assistantships

We grouped explanations from individual participants by learning process (Mechanism) and thus identified context-mechanism-outcome (CMO) configurations, i.e. what it was in the context which had triggered this mechanism and what they had identified as the outcome in terms of preparedness. Recurring CMO configurations formed ‘middle range theories’ explaining what students and junior doctors perceived as equipping students for practice and where the gaps are in UK medical school curricula. These middle range theories were formulated by discussion between all researchers and are explained with illustrative quotes in the results section. They are then drawn together into a ‘programme theory’ of elements of the assistantship which are important for developing entrustment and in turn preparing students for practice (A visual representation of this is offered in Fig. 2 in the discussion section below).

### Reflexivity

We are a multidisciplinary team, with a range of research expertise and experience relevant to the methods used in this study, comprising clinical academics (three general practitioners, a palliative care physician and an emergency physician) and a medical student. All but SY are stakeholders in the Keele curriculum having developed and led aspects of the program since its inception or through direct student experience. We brought the perspectives of our disciplines to the development of an initial programme theory. We rigorously tested our individual explanations in group discussions to ensure that all perspectives were examined in the light of our disparate gazes.

## Results

### Participants

Thirty-two final year students completed log sheets. Twenty-seven of these also kept audio diaries, one kept a written diary and 24 were interviewed. Fourteen of these participants consented to be followed through their transition to junior doctor. Eleven of the 14 submitted audio diaries and six of the 11 were interviewed. In addition, 57 junior doctors from three local hospitals who were graduates of 17 UK medical schools participated in focus groups and interviews (see Table 1 above).

Our results demonstrate that during the assistantship students logged more EPAs than non-EPAs though discussion about clinical activities with supervisors, even when not directly participating in an activity, was also important to learning (see Table 3: Appendix). Examples of activities logged by students—classified by Utrecht EPA type—demonstrated engagement with a broad variety of junior doctor-type activities (Table 3: Appendix). We have grouped actual activities which students reported undertaking and explored the meaning made of and learnings taken from them retrospectively. No students described activities they reported as being other than constructive to learning. Non-EPAs are also listed in Table 3.

#### CMO configurations containing EPAs

Our data from graduates of 17 UK medical schools suggests that, although none are using EPAs as units of assessment, participants had engaged with a variable range of EPAs as undergraduates. This varied by type of course; type of placement; type of activity. We identified recurring CMOs containing EPAs which differed between schools and placements. Configurations of context mechanism and outcome (CMOCs) containing EPAs are summarised (emboldened text) and each set of CMOCs is illustrated with quotes and developed below:

## The student as a team member

*A culture where the student is seen as a team member (context) enables access to supervised practice with FY1 jobs (mechanism) which is an important feature of students and graduates feeling prepared for practice (outcome).*

Allowing students to become participative team members was perceived as important in facilitating access to supervised opportunities and, in turn, being acknowledged as making useful contributions to clinical work. Both students and new doctors described how being supported to complete EPAs ahead of graduation provided more effective learning than observation. Students able to utilise the available opportunities to demonstrate their clinical capability (usefulness) to their clinical supervisors also reported a feeling of being admitted to the team and with that, receiving permission or feeling confident to undertake further professional tasks, whilst also learning how to draw on team support.

Student Interview ID17m:* you’d start with sort of bits and pieces like that and then obviously as they realise that you are actually useful and not entirely useless to them, then you begin to pick up other bits and pieces. It depends where the FY doctors you are based with came from, so if they were Keele graduates, quite a few of them understood the idea of the sort of fifth year role and were quite keen to say okay, you do all the note-taking for this ward round, for example, or you do this first patient and I’ll do the next one and so on and so forth, to try and get you into the hang of it.*

Students and doctors talked about the benefits of belonging in a team and having sufficient time on a placement to develop team relationships. Nevertheless, while the medical schools may intend that students join and learn within teams, students' experiences may fall short of that experienced by graduates due to the inherent change in responsibility and identity associated with employment.

Site 2: FG 1 IDM11 (doctor):* yeah, despite sort of clearly strong attempts by medical schools to make sure that fifth year is the time when you start to feel integrated as part of the team with longer rotations, I think there’s just something inherent in working as part of that team as opposed to sort of being a visitor or observing that team.*

Whilst doctors also perceived that being given FY1 ‘jobs’ is good preparation for practice, it was clear that the duration and scope of practice experienced during placements varied between medical schools with participation ranging from observation of FY1 activities to comprehensively integrated assistantship. Graduates of schools where placements were shorter felt insufficiently prepared and disadvantaged.

FG2U (doctor):* I’ve been with a Keele graduate, [name], and she seems really prepared. I think… I think from what I understand, that 15 weeks you do as an F1, I think is a really good thing and I think it’s a shame that… we didn’t have it.*

FG4St(doctor):* cause when I came and I was hearing that people had been doing this for months now, and I was like well, I’ve done this for 4 weeks and I’m starting literally as if I’m already behind everyone else, so it affected my confidence.*

However, this could be offset by the student’s individual initiative, an important internal context (internal): since more engaged (mechanism) students get to do more and feel better prepared (Outcome) – see FGS1-M12**(doctor)** in Legitimate activity.

## Legitimate activity

*A shared understanding of what constitutes important assistantship activity (context) triggers the initiation or gate keeping of engagement with clinical activities (mechanism) and is important in the development of professional capability (outcome).*

Important contextual factors are both internal and external to the assistantship. They include undergraduate institutions giving priority to placements over other activities such as summative examinations, competing learning activities and whether student engagement with clinical activities is considered legitimate with regard to legal considerations, local policies, social norms and hierarchies.

Entrustment to perform clinical activities, whether at (Utrecht) level 1, 2 or 3 supervision, is usually initiated by the clinical supervisor rather than the student, though motivation and self-identification as a proto-FY1 (FY0) (student’s internal context) is also a facilitating factor.

FGS1 M12(doctor):* it was… I found …. at xxxx when I was a student, like you know, no one would know when I was turning up, I would go and just ask the juniors if there was anything I could do, you know, and they were rushed off their feet and because I was turning up at sort of ad hoc times, they didn’t really know who I was and stuff – there was no continuity. So I think, yeah, I think there’s such a better way to use students….*

When there is not a local arrangement that students will be involved in an ‘FY0’ role, more junior doctors are not always aware of intended learning outcomes for students, and what students can or can’t do.

Medical schools may stipulate that students be accepted into clinical teams and encouraged to participate in clinical care. However, unless it is communicated how this should work in practice, including translation into meaningful units of activity the clinical team may not support engagement. Where it was achieved it was viewed as outstanding.

FGSt4(doctor):* my favourite firm at med school was my last medicine one – it was only four weeks, but the consultant was incredible – and I think it’s … not depending on (your) medical school… It’s the consultant that’s in charge of you. He was like “right, when I’m on call, you’re on call. When I’m on take, you’re on take”, and he saw us from the start and he saw us to the end and like knew what our weaknesses were, what our strengths were, and helped us develop them.*

Where the team doesn’t deliberately involve students, students may not push themselves forward. The reasons for this may not be apparent to the supervising clinician and may be viewed as disinterest.

CT2 FG1(doctor):* because what I’ve noticed with students who are coming onto the ward, they are simply observers, they do not get involved, you know, the consultant says “what do you think about this x-ray” or “what do you think about this thing” – they do not get involved, they just simply observe, follow you, they don’t say “oh, can I just read up the obs” or “can I write in the notes” or “can I do this” – they don’t volunteer unless you say “would you like to do that” or “do you mind to read the observation chart” or “do you mind to have a look on the medications to see what they are taking”. So sometimes I think that… because they don’t engage so much, or they don’t want to get involved – either they are afraid or I’m not sure what their reasons are.*

Curricular design is critical to successful engagement with the assistantship. Shadowing needs to be at the right time, after final knowledge assessment, in order to avoid distraction and to capitalise on the opportunity to apply learning.

FGS1 M12(doctor):* ‘cause we only had about four days, of which half was filled with induction, so it’s like a problem when you actually sort of start, but I do think… you know, I did my shadowing two or three months before my finals, and I barely went in ‘cause I was studying, so you know that wasn’t great for me in terms of my learning in terms of the job I was going to be doing. But we did do one month shadowing straight after finals, which was actually quite good because we didn’t really care about learning stuff, it was just doing the F1 jobs and that was really useful.*

It is important to align assistantship learning with the new doctor role. During the GP assistantships there is no immediate junior doctor role model available and therefore the student was not always able to appreciate the relevance of their activity. However, there was still talk about learning the many tasks of FY1 (outcomes) by ‘picking up’ (mechanism) which seems to involve observation and probably discussion of letters from FY1s (level 1) and doing (level 2 or 3).

Student interview ID15f:* little things that I’ve picked up – so how to write TTOs (discharge medications), the good and bad discharge letters which I learned from GP, knowing about doses for common drugs, and just how to use the computer systems to order things and where to take x-ray requests to and things like that really.*

Juniors are still “frightened” of sick (acutely unwell) patients and would welcome more exposure to these patients in undergraduate programmes.

FG2U(doctor):* that’s what a lot of the bosses at the hospital are worried about, is us not being safe to pick up the people who are really unwell or having seen them… I think if we’d done a couple of weeks as a student on SAU a couple of weeks on AMU, it would give you the… like the insight into this patient really needs help, this patient doesn’t. I’m not going to say we’re going to be 100%, but… it’d just give us a bit more confidence.*

Being made to do out of hours/night shift as a student (context) was felt to be important as it was hoped that it would make FY1 night shifts less of a shock (outcome) and help to develop coping strategies (outcome).

Student ID12m:* having spoken to a few of the people that were the juniors there that had been training at other medical schools, who said that, you know, they’d never been expected to do a night shift once, so obviously when they first had to do their first night shift it was a massive shock to the system, but having already had the experience of that shock then I should be better set and better prepared for it and I’ll know when I’m starting to experience issues and I’ll know when I need to start almost trying to think more and being a lot more careful with what I’m doing.*

Doctors agreed it was important to do the nights on call but in order to promote this type of learning and to be authentic a post night shift recovery time was required. It was also expressed that even night shifts were not truly authentic F1 experiences because students were never left unsupervised when performing clinical duties.

## Practice makes perfect

Providing opportunities (context) for repetitive practice (mechanism) enhances speed, accuracy and confidence of the junior doctor (outcome). Undergraduate preparation for junior doctors’ EPAs (outcome) should be supported by dialogue and feedback (mechanism). Where clinical practice cannot be directly supported, role play (mechanism) and discussion (mechanism) are also important feature of EPAs. This is particularly so for EPAs which are not readily available to students and is at least part preparation for coping with them as a doctor (outcome).

Performing clinical procedures as a junior doctor is common and was one of the most frequent activities logged. Though it is now the norm that students are taught procedural skills in simulation and undergo capability assessments prior to clinical practice they appreciate level 2 supervision and feedback when attempting to perform clinical procedures (mechanism) in clinical practice (context), in order to refine skills (outcome) and avoid patient harm (outcome).

*Student audio diary ID05*:*Not had much practice with IM injections since year 2/3. The way I did it was rather clumsy. Had I not caught myself at the last moment (with the doctor supervising me also reminding me about that last step of the procedure at the same time) this may have been a potential harm to the patient.*

Performing a procedure in vivo for the first time as an FY1 is difficult. Locally, interventions such a procedural skills logbook, were perceived to drive learning. Achieving competence with procedural skills also requires repetitive practice (mechanism).

*Student 17 m*: *learning by doing – is really beneficial …take the example of, say, cannulation – if I’ve been told about it and seen it and things like that, it’s all very well and good but once I’ve actually done it, I find that sticks a bit more in my mind.*

However, interventions vary between schools: preparation is seen as being patchy, each school having its own strengths and weaknesses.

*Keele graduate ID31f voicemails (docto*r): *we needed to certify my blood taking ability and also certify my transfusion ability and I was surprised that one of the other girls from one of the other medical schools actually hadn’t done that before, so I felt generally quite well-prepared and that actually she’d only ever used butterfly needles in her hospital, which I thought was quite interesting.*

Interventions such as the skills passport were also criticised for forcing students to seek opportunities to demonstrate competence (mechanism) in a set of procedures that they may not do as FY1s (perverse outcome), while not experiencing or completing real FY1 tasks (missed outcome)**.** Participants felt that other activities, such as ward rounds, extraordinary care and prescribing, were also learnt better in practice than by other methods.

*Student interview ID15f*: *I’d previously tried different ways of learning drugs and their classes and their doses, and I knew it for exams, and then two weeks later I’d forget it. But I’ve actually found that actually doing it and prescribing was the way for me to learn them.*

Providing ‘authentic’ experiences of common FY roles and responsibilities e.g. ward rounds, attending cardiac arrests (context) adds value and aids the transition by habituation (mechanism), knowing what to do but also feeling confident about doing it (outcome).

*Keele graduate audio diary ID15f (doctor)*: *The ward round was fine, that was not a problem, and I didn’t feel really any different to that as a final year medical student – it was the same sort of role – the only difference was—as going round, if patients needed any new antibiotics prescribing or fluids, I could do that, and I felt confident in doing those prescriptions.*

Discussion appeared frequently in the student learning logs referring both to discussion of EPAs and non-EPAs. Discussion (mechanism) was felt by learners to be an important adjunct to participating in EPAs.

Student Interview ID27f.

Interviewer: *which learning method do you think you were doing most of?*

*Probably discussion, I would have said because a lot of my clinics are done by myself, so with a supervisor but they’re running their own clinic as well – so it’s like a parallel [clinic] – once I’ve seen my patient, I tell the doctor I’m ready and then they come and then it’s a very kind of, I do a quick summary of what the history’s been and then we… you know, they come up… if I can, I come up with a management plan and then they either agree or they come up with a management plan if I’m not sure, and then it’s very quick and we kind of, right that’s it, send the patient away, and then we might have a quick discussion then and then they leave and I bring in the next patient.*

Where opportunities for supervised clinical practice were more limited (context), such as in the assessment of emergencies or giving extraordinary care (outcome), participants reported the value of learning through simulated practice (mechanism), discussion and feedback on their performance (mechanism).

## Interview FY1 2 m (doctor)

*At [hospital name] we had two or three full-day sim sessions where they just had emergencies – severe asthma coming in, upper GI bleed, sepsis, goes into arrest – and we also had a few here, we had sepsis goes into arrest here that I was part of. …. I don’t really deal with emergencies, those are my only exposure to them, so they’re pretty invaluable for nailing down what you should be doing and how you should be doing it – they give you good feedback and it gives you a chance to try it out which is wonderful.*

Our final programme theory combines these three middle range theories and explains how student EPAs can prepare them for the transition (Fig. 2).

## Discussion

Our results demonstrate the potential for plentiful access to EPAs during final year medical assistantships. The majority of naturally occurring EPAs were classified as either “The Clinical Consultation” or “General Medical Procedures”. Access to EPAs was determined by context including placement location, a culture that fosters student team membership and curricula that align learning with the new doctor role. Primary and secondary care placements complemented each other providing more consultation based and more practical procedure-based EPAs respectively. Students reported relatively little experience of EPAs involving extraordinary patient care, perhaps surprising given the higher acuity of patients in secondary care settings. As noted previously, when students did experience extraordinary care their level of entrustment was often limited to observation, discussion or simulation (Cutrer et al., 2019). However, students still valued and logged this learning supporting the importance curricular alignment and recognising the value of low-level entrustment ahead of practice. Unsurprisingly, such activities were frequently cited as ‘firsts’ during the post-graduation transition period (Lefroy et al., 2017).

Our initial programme theory combined our empirically derived mid-range theory from the first paper (Lefroy et al., 2017) with the learning theory that underpins EPAs (Ten Cate & Scheele, 2007) In our final programme we have refined this theory, adding flesh to the bones about how high level CMOs relate to EPAs and specifically to explain how assistantship-type placements can act to support access to EPAs and the development of higher levels of entrustment (Fig. 2).

Access to EPAs requires acceptance of the learner by the clinical team and the learner’s participation in the team’s work. However, students report finding themselves relegated to the periphery of patient care (Kuehl & Spicer, 2022). Assistantships redress this by advocating shared responsibility for patient care (GMC, 2022). Supporting a student to access a clinical task requires trust and creates interdependence and vulnerability for both parties. Trust occurs variably and is a product of the characteristics of the trainee, the supervisor, the supervisor-trainee relationship and the context (Caro Monroig et al., 2021; Sterkenburg et al., 2010). Initial trust, based on first impressions, is replaced by grounded trust as developing competence is demonstrated (Tekian et al., 2020). Access may be improved by providing credentials (evidence of previous capability) as it fosters presumptive trust (Ten Cate et al., 2016). Being trusted and supported to complete EPAs gave students a sense of being in the new doctor role, important for building confidence and self-efficacy (Bandura, 1984; Braniff et al., 2016; Dornan et al., 2007; Eraut, 2004; Monrouxe et al., 2018). Successful assistantships were described in active terms (acting as an FY1) rather than passive (shadowing). Professional identity formation (PIF) describes the process by which medical students develop the knowledge and skills they need to demonstrate professional behaviour (Cruess et al., 2015). EPAs are activities that medical students can and are expected to perform. They stimulate participation in delivering care and help students take responsibility for their own role (Bremer et al., 2022; Caro Monroig et al., 2021). With developing trust students find it easier to access activities, feel better able to show their development needs and seek support from the clinical team, thus supporting their relational identity development (Cruess et al., 2015). The opportunities to tackle clinical tasks are triggered when supervisor and student have a shared understanding of what constitutes legitimate assistantship activities (Pinilla et al., 2021) and students are empowered to recognise and share their shortcomings (Caro Monroig et al., 2021). Where guidance is provided about a student’s capability it is well received and aids placement decisions about acceptable engagement (Tekian et al., 2020). It is also possible that EPAs themselves might augment clear communication of the degree of entrustment and the level of offered or received responsibility between supervisor and student, adding to their own educational value still further.

Placements facilitating the building of longitudinal, as opposed to shorter-term relationships, permit more engagement in clinical care (Caro Monroig et al., 2021) We also identified that junior doctors who had prior personal experience of assistantship placements engaged students in EPAs more readily and were viewed as role models. This powerful, unconscious patterning of behaviour aids the drawing-in of the learner from the periphery to the centre of the community of practice, and the acquisition of tacit knowledge which itself aids the development of professional identity (Cruess et al., 2015).

Students’ sense of preparedness and transition into graduate practice was enhanced by repetition of activities and feedback. However, whilst the primary care assistantship was a rich source of consultation focused EPAs and feedback, students did not always see the immediate relevance to the junior doctor role. Some students expressed anxiety about being out of the hospital environment just before graduation and specifically cited concerns about performing medical procedures and their paucity of recent experience.

EPA frameworks aim to clearly delineate the skills student should possess at graduation, medical students involved have yet to recommend their inclusion as a graduation requirement (Geraghty et al., 2021). Our previous research suggests that preparation for practice can never be complete because of the step change in responsibility which comes with adoption of the identity of a junior doctor (Lefroy et al., 2017). However, trainee participants were able to offer insights about activities that represented ‘firsts’ once qualified or that they struggled with where preparation might be improved. Defining the professional activities that an undergraduate should be encouraged to participate with, even at the level of observation, aids engagement. With successful completion of simple tasks, trust develops and with it access to more complex tasks and support with learning. This supports individual professional identity development through participation, since the student begins to think, acts and feel like a junior doctor (Cruess et al., 2016). Integration and socialisation within the team helps students mature in terms of their relational development and enables them to move from peripheral towards more full participation in essential activities of care (Vivekananda-Schmidt et al., 2015). Assistantship placements have the capacity to provide a legitimate role for shared responsibility, time to build relationships with the clinical team and the student’s agency to engage. Professional agency, being proactive towards work, team safety, personal development, is believed to be enhanced through longitudinal student-supervisor relationships (Bonnie et al., 2022; Caro Monroig et al., 2021), supporting assistantship placements of longer duration.

### Strengths and limitations

We used Utrecht’s classification of EPAs for undergraduates (Ten Cate et al., 2018). While simpler than others (Sohrmann et al., 2020), it has been validated and their simplicity brought clarity to the coding which outweighed the potential for coding more of our students’ learning activities as EPAs using more complex schedules. Our participants’ talk about preparation was extracted from a data set which had been rigorously coded for our previous studies (Lefroy et al., 2017).

We acknowledge that despite our efforts to maximise the immediacy of the student log books and reflective diaries by sample for one week at a time rather than more longitudinal sampling, engagement with the learning logs was variable: for example, some students logged individual consultations, others logged whole clinics or ward rounds as a single learning activity. However, we coded one entry whether a consultation or a clinic as one activity so we are likely to have underestimated the number of EPAs our group of 32 students encountered during their six weeks of logging activity. Furthermore, we acknowledge that our data on preparation for practice by the 17 other schools is retrospective but note that it closely resembles findings elsewhere (Monrouxe et al., 2017). We also acknowledge that we have combined data from very different sources (near contemporaneous logs and reflective diaries, stimulated recall interviews and reflections aided by those records, and interviews and focus groups inviting retrospection over the eventful years of early practice). However, realist evaluation is particularly suited to the synthesis of disparate data.

## Conclusion

Final year medical students are engaging to varying degrees with EPAs. As has been described elsewhere, self-directed learners who more actively engaged with ‘EPA like’ activities reported more enhanced preparation for clinical practice through legitimate participation and engagement with the work of the team (Caro Monroig et al., 2021). This in turn supports professional identity formation through the opportunity to take on the professional role and ‘act like a doctor’ and socialisation by being made to feel ‘part of the team’ (Cruess et al., 2015; Vivekananda-Schmidt et al., 2015). Synergy in understanding what constitutes important EPAs appears to trigger access to EPAs at an appropriate level of supervision. Engagement is dependent on context and relevant context includes placements that provide a legitimate role in the team providing access to relevant duties, supervisors attitude to actively engage student in EPAs (Stephan et al., 2023) and the supervisor-student relationship that must support a student’s sense of agency to ensure appropriate level of supervision (Caro Monroig et al., 2021). Assistantship placement can enhance this process if the curriculum explicitly validates learning in the clinical context and frees students to do so rather than appearing to focus on knowledge acquisition and testing (Crossley & Vivekananda-Schmidt, 2015). Placements also need to be of sufficient duration to allow the development of grounded trust between the learner and their clinical supervisor (Bonnie et al., 2022). We suggest that these seemingly little things are actually big things (and why) when it comes to students being able to engage in the assistantship activity that is important to their development of preparedness. Enabling these contexts through developing techniques specifically designed to support a shared understanding between student and supervisor may result in an increase in the amount and/or quality of important EPAs that take place and is worthy of further investigation.

## Appendix

See Table 3.

**Table 3:** Examples of activities logged by students classified by Utrecht EPA type

The clinical consultation	
• Primary care	• Secondary care
• Parallel consulting with discussion of each case (e.g. unwell children, acute psychosis, low back pain, assessment of tiredness, threated miscarriage, assessment of memory problems)	• Assessed patient with phlebitis from a cannula
	• Examined patients on the ward round, presented findings
	• Ordered tests/investigations
• Co-consulting with GP	• Assessed (clerked) acute admissions and commenced immediate care
• Joint home visits	
• Prescribing practice with GP	• Discussed patient management with doctor
• Assessed a patient with dyspepsia and referred to urgent clinic	• Assessed patients whose condition had deteriorated
• Performed a contraception review	• Assessed and presented findings of patients seen in Colorectal clinic
• Completed a triadic interview	
• Assessed a patient with abnormal blood test due to a drug interaction	
General medical procedures	
• Primary care	• Secondary care
• Gave a steroid joint injection	• Intravenous cannulation
• Collected vaginal swabs	• Venesection (including sampling from femoral vein)
• Measured blood glucose	• Commenced intravenous fluid infusion
• Inserted intrauterine device	• Collected blood cultures
• Administered hormone injection	• Arterial blood gas sampling
• Inserted subcutaneous contraceptive implant	• Inserted urinary catheter
• Completed minor surgical procedures	• Administered subcutaneous injection
• Venesection	• Inserted a thoracic drain
• Administered intramuscular injection	• Fluid bolus administration
• Performed an electrocardiogram	• Recorded vital signs (e.g. blood pressure)
• Performed spirometry	• Assisted with a blood transfusion
• Measured Ankle-brachial pressure index using a Doppler	• Assisted with wound care
• Performed a glucose tolerance test	• Prepared and administered intravenous antibiotics
	• Observed induction of anaesthesia (unstable cardiac patient)
	• Performed airway management including tracheal intubation
	• Taught vascular examination (using US Doppler) to junior students
Informing guiding and advising patients and families	
• Primary care	• Secondary care
• Explaining diagnoses	• Observed breaking bad news on ward round
• Advised patients about their medications	• Palliative care consultation with patient
• Explaining use	• Communicated with a family about the progress of a patient
• Medications to avoid in pregnancy	
• Chronic disease management e.g. adjusting insulin dose	
• Cessation of drugs due to occurrence of complications	
• Performed annual diabetic review	
• Constructed personalized patient plan (COPD)	
• Gave preventative health advice (e.g. alcohol, smoking cessation, diet)	
• Explaining radiological and blood test findings	
• Observed the use of sick notes	
• Observed negotiation with a patient – use of investigations	
Communicating and collaborating with colleagues	
• Primary care	• Secondary care
• Co-consultation with advanced nurse practitioner	• Ward round duties: Made entries in patient records, ordered investigations, followed up test results
• Managed an acute case jointly with the paramedics	• Wrote discharge letters—concisely summarised a patient’s inpatient care and checking their medication charts
• Presented the results of an audit of antibiotics at the practice meeting	• Induction—Helped orientate a new member of the team (FY1) to the ward
• Assessed and presented the case of an elderly patient requiring further support. Was advised how to refer for social care	• Completed specialty referral forms
• Palliative care in the community	• Made telephone referrals
• Read hospital letter and completed actions and follow-up requests	• Requesting advice from on-call registrars (with support from doctors)
• Taught a more junior medical student	• Handing over information to nurses
	• Reviewed blood results and made management decisions with the support of weekend FY1s
	• Organized the transfer of a patient to the radiology department
Extraordinary patient care	
• Primary care	• Secondary care
• End of life care day	• Observed registrar managing a peri-arrest patient
• Home visit to terminally ill patient	• Cardiac arrest, ABCDE assessment and peri-arrest management
	• Verifying death
NON-EPAs logged by students	
• Primary care	• Secondary care
• Patient survey	• Radiology teaching
• Audit project	• Had careers discussions with doctor colleagues
• Reflective writing	• Attended postgraduate teaching (for FY1 doctors)
• Weekly Seminars	• Attended the hospital Grand Round
• Portfolio & CV development	• Coroner’s court
• Types of hearing aid + communication with deaf patients	• Discussion of death certification with doctor
• Prescribing workshop	• Teaching on ECG interpretation
• Discussion of career in GP	• Applied ethics casework and teaching session
• Discussion of when to submit an incident report	• Professionalism workshop
	• Portfolio and CV development
	• Preparation for the national prescribing safety assessment

Generate a list of firsts for the FY1 as a warm-up exercise (flipchart, this will be.source material for the FY1 audio diary study next year).

- What is it like doing things for the first time?
- How has reality compared to your anticipation of transition?
- How did your medical school experience prepare you for these transitions? (what

transitional doctor roles were you given, what activities were helpful?) Consensus?

Different views? (this will be source material for the Y5 study)

- Relational firsts?

Three types of firsts to ask about:

1. One or two which they have mentioned being significant for them
2. Those which surveys show FY1s feel poorly prepared for (eg prescribing)
3. Some of the firsts we listed which they may have done already as a med
4. student (eg writing TTOs and discharge summary) so they can talk about the
5. transition having started in med school (if it did)

What might you have done at medical school which would have helped you make the.transition? Generate a list of roles/activities. (This will be source material for the Y5study)

- What else has helped you with the transition? Consensus? Different views?

- What have you found difficult in the transition from student to doctor? What gives you.confidence? Consensus? Different views? Discuss differences betweencompetence / confidence / comfortableness.

- Learning environment? Learning in practice?

Hand round or put on a screen the preparedness questionnaire data for fresh.graduates of UK med schools and ask the participants to recall their own feelings ofpreparedness at that time. Consensus? Different views?

- Why are there differences between medical schools in the levels of preparedness of.their graduates?

- What do you think about 15 weeks in GP as preparation? 5 weeks in critical care? 5.weeks in medicine? 5 weeks in surgery? (This will also link with Y5 study)

- Being the GP on the wards?

*NB List of “firsts” we are thinking of, to be added to by the group interview with.*

*FY1s:*

- Death of a patient in your care; Confirming death.

- Crash call.

- managing the acutely ill patient.

- managing the various patient transitions at the end of life.

- Prescribing (there may be a number of firsts within this).

- Error.

- Completing a discharge summary.

- Completing a death certificate.

- Phoning the coroner.

- Night duty.

- Requesting an investigation.

Interview schedule

*Prior to the interview read through the student’s 5 day learning log and listen to their audio diary or read the transcript. Note any points which you think require clarification or are interesting for exploration especially to do with the way they are learning and their anticipation of transition.*

Intro: Information was made available and consenting was done on line, so it may have faded in your memory. Do you have any questions about the process before we start? Interviews will be audio-taped and transcribed in an anonymous format so that they can be used by the research team. You should feel free to say whatever you want and I will not raise it with you should we meet again.

Questions about Learning:

- What do you feel is the main aim or aims of this assistantship? And of year 5 in general? (*students may be serving several masters or learning for goals which are not necessarily aligned: to pass final examinations; to be able to function as an FY1 doctor; to prepare for a career in specialist medical practice*. Please try to ascertain which of these are active for this student now)
- You have kept a log for a week and categorised your activities by method of learning: (Observing /Doing /Listening to a talk or Reading about/ Discussing)
  - Which method are you doing most of,
  - which do you learn best from
  - and which would you say is your favourite and why?
  - Would you like the balance to be different between these?
  - Please can we clarify what you mean by xxx(with line references) in your learning log and xxx(with line references) in your audio diary?
  - Please can we explore your comments about xxxx (with line references) *(we are especially interested in roles, relationships, methods of learning)*
  - How much do you feel you are “part of the team” as a senior apprentice? What makes / would make you feel part of the team? Is it important to be part of the team?

Questions about Transition:

- How do you feel about the transition to being a doctor?
- Please can we explore your comments about xxxx (with line references) *(if there were any comments about anticipation of transition)*
  o How do you think it will be different to being a final year student?
  o What do you think will be the most significant ‘firsts’?
  o What are you most looking forward to?
  o What are you least looking forward to?
  o Can you describe how you think about what your responsibilities and role in the team will be?
  o What about your interactions with other healthcare professionals?
  o How do you feel about prescribing?
  o How do you feel about seeing acutely unwell patients?
- How is your medical school experience so far preparing you for these transitions? (what transitional doctor roles are you being given, what activities are helpful?)
- What else might you do at medical school to help you make the transition? *When they have said what they want to, ask them about their views on the roles/activities suggested by the FY1 focus groups:*
  o Weekend phlebotomy
  o Physician's assistant—students to shadow FY1 and their shifts (1 month after the FY1 post starts)
  o Out of hours experience

Have you already done any of the following FY1 tasks?:

- Have you any experience of attending crash calls?

- What about holding a bleep?

- Have you been given the task of breaking bad news?

- Have you requested investigations? Out of hours?

- What do you think about

- 15 weeks in GP as preparation? (What do you learn there which will help you in FY and beyond?)

- 5 weeks in critical care? ditto

- 5 weeks in medicine? ditto

- 5 weeks in surgery? ditto
